# Selection and Evaluation of Candidate Reference Genes for Quantitative Real-Time PCR in Aboveground Tissues and Drought Conditions in *Rhododendron Delavayi*

**DOI:** 10.3389/fgene.2022.876482

**Published:** 2022-04-14

**Authors:** Lu Zhang, Yanfei Cai, Mingchao Zhang, Guanghui Du, Jihua Wang

**Affiliations:** ^1^ Flower Research Institute of Yunnan Academy of Agricultural Sciences, Kunming, China; ^2^ National Engineering Research Center for Ornamental Horticulture, Kunming, China; ^3^ School of Agriculture, Yunnan University, Kunming, China

**Keywords:** gene function, genomics, housekeeping gene, marker-assisted breeding, transcriptomics

## Abstract

There has been no systematic identification and screening of candidate reference genes for normalization of quantitative real-time PCR (qRT-PCR) results in *Rhododendron delavayi* to date. Therefore, the present study used *GAPDH, Act, EF1, Tub-, Tub-5, UEC1, TATA, TATA-2, UEP, TIP41,* and Ubiquitin to predict their stabilities on different aboveground tissues (matured leaves (ML), stem tips (STM), and flower buds (FB)) at different developmental stages (young and adult plants) using five statistical algorithms: Delta Ct method, BestKeeper, geNorm, Normfinder, and RefFinder. The findings were confirmed using ML obtained from plants that had been stressed by drought. By using RefFinder with ML samples collected under drought conditions, it was determined that the top five most stable reference genes were *GAPDH > UEC1 > Actin > Tubulin- > Tubulin—5*, whereas the least stable reference gene was Ubiquitin. In addition, under control conditions, *UEC1, UEC2, Actin, and GAPDH* were selected as the highest stable potential reference genes at the juvenile stage of *R. delavayi* with ML and STM. When ML and STM were combined with drought-stressed samples, *TIP41, GAPDH*, or their combination proved to be the most effective qRT-PCR primers. The findings will aid in the improvement of the precision and reliability of qRT-PCR data and laying the groundwork for future gene functional studies in *R. delavayi*.

## Introduction

*Rhododendron delavayi* Franch possesses very attractive flowers and good resistance to arid and cold climates contributing to a high demand in the Chinese market and other Southeastern Asian countries, such as Burma, India, Thailand, and Vietnam (Zhang et al., 2017). It has medicinal properties such as antioxidant, anti-inflammatory, anticarcinogenic, and antibacterial properties making it a suitable candidate for the treatment of diabetes, arthritis, headache, and hypertension (Cao et al., 2004; Qiang et al., 2011; Zhou et al., 2014). *R. delavayi* is widely distributed and cultivated throughout southwest China and grows at a wide altitudinal range between 1,200 and 3,200 m (Ma, 2015; Zhang et al., 2017).

The draft genome of *R. delavayi* was published in 2017 by Zhang et al. (2017), and since then, a number of molecular studies have been conducted on this species (Cai et al., 2019; Sun et al., 2021). For instance, Sun et al. (2021) functionally characterized dihydroflavonol4-reductase–designated *RdDFR1* and revealed its involvement in flower color formation in *R. delavayi*. In addition, Cai et al. (2019) performed physiological and transcriptomic analyses to identify key metabolic pathways involved in drought tolerance in *R. delavayi* and further conducted quantitative reverse transcription polymerase chain reaction (qRT-PCR) with ten differentially expressed genes detected from the prominent Kyoto Encyclopedia of Genes and Genomes (KEGG) enriched pathways.

Omics, such as genomics and transcriptomics, and their application have been essential in revealing several key genes and metabolic pathways modulating numerous traits of agronomic and medicinal importance and aiding marker-assisted breeding in crop species (Channale et al., 2021; Cui et al., 2021; Maritim et al., 2021; Xiao et al., 2021; Zenda et al., 2021). One of the requisites for genomics and transcriptomics is qRT-PCR which measures changes in gene expression (Phillips et al., 2009; Köhsler et al., 2020). The prominence of qRT-PCR is due to its precision, accuracy, convenience, speed, and sensitivity (Wang et al., 2016). However, qRT-PCR requires a housekeeping/reference gene to act as an internal control to normalize the expression level of target genes (Bustin et al., 2009; Gao et al., 2017). Preferred housekeeping genes must be stably expressed in organisms under varied biotic and abiotic conditions (Zhu et al., 2014) and in different tissues (Janská et al., 2013) but not affected by the stage of development of the organism (Huis et al., 2010) and endogenous or exogenous factors (Janská et al., 2013).

The previously mentioned background suggests the need to screen and select appropriate reference gene(s) to accelerate the breeding process in *R. delavayi*. A number of reference genes for qRT-PCR have been reported to include *glyceraldehyde-3-phosphate dehydrogenase* (*GAPDH*), *actin* (*Act*), *ribosomal protein S3* (*RPS3*), *elongation factor 1-alpha* (*EF1α*), *tubulin-beta* (*Tub-β*), *TATA binding protein (TATA), Ubiquitin-conjugating enzymes E2-28 and E2-32* (*UEC1 and UEC2*, respectively), and many others (Fan et al., 2013; Gao et al., 2017; Jin et al., 2019; Yang et al., 2019; Li et al., 2021). To the best of our knowledge, there has not been any report on the selection and validation of reference genes in *R. delavayi*; therefore, the present study used eleven well-known reference genes: *GAPDH, Act, EF1α, Tub-β, Tub-β5, UEC1, UEC2, TATA, UEP* (Ubiquitin domain–containing protein), *TIP41* (TIP41-like family protein), and *Ubiquitin* (Ubiquitin-40S ribosomal protein S27a) to profile their expression in different tissues at varied developmental stages under control and drought conditions. In addition, we utilized a well-known gene– 9-cis-expoxycarotenoid dioxygenase1 (*NCED1*) involved in water stress (Changan et al., 2018) to study their stabilities under drought conditions either individually or combined tissues with five statistical algorithms: Delta C_t_ method (Silver et al., 2006), BestKeeper (Pfaffl et al., 2004), geNorm (Vandesompele et al., 2002), Normfinder (Andersen et al., 2004), and RefFinder (Xie et al., 2012). The results would provide valuable information and lay the foundation for further functional validation of gene expression by qRT-PCR and biosynthetic pathway and metabolic engineering in *R. delavayi*.

## Materials and Methods

### Plant Materials, Growth Conditions, and Drought Stress Treatment

**FIGURE 1:**
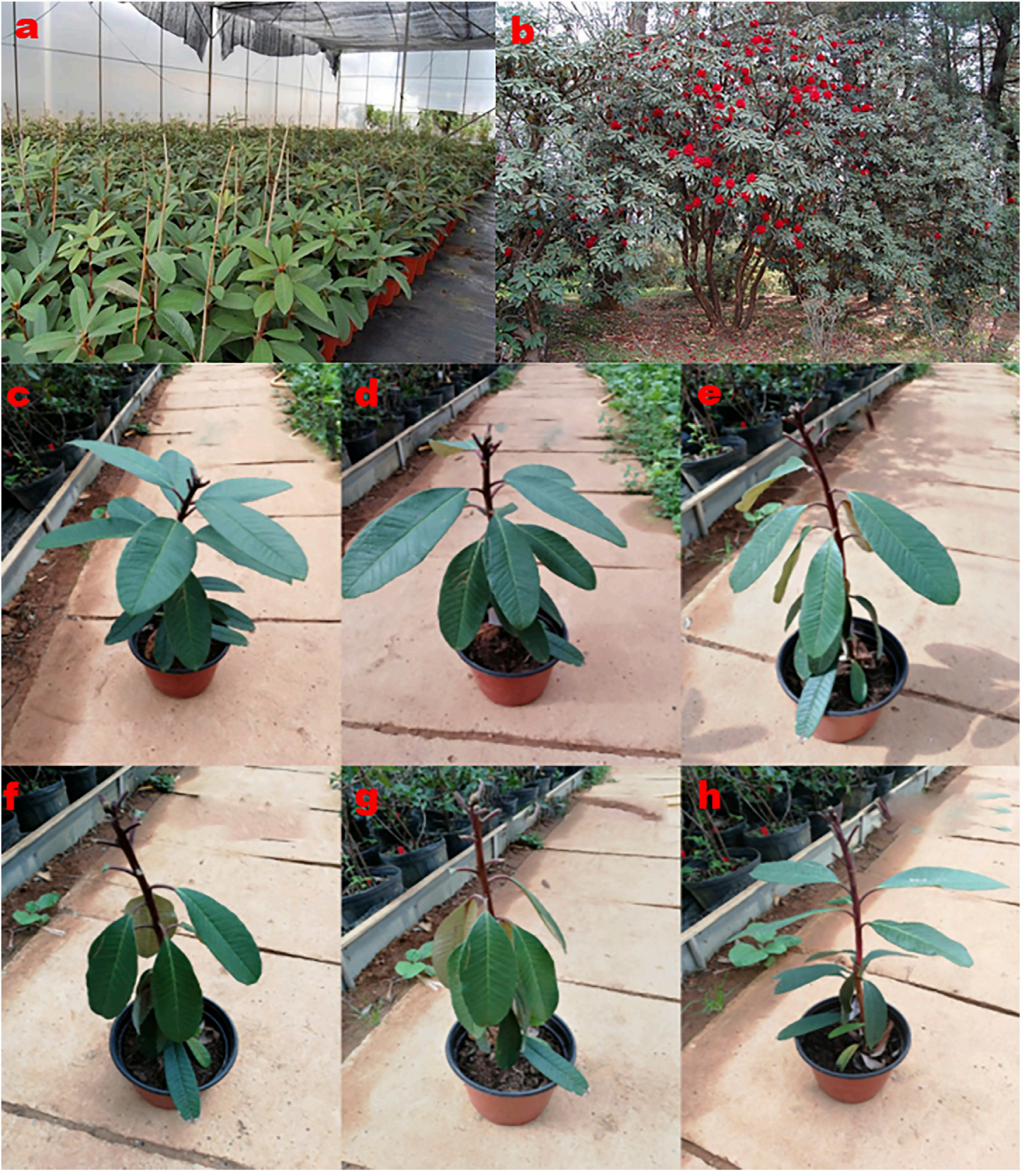
Samples of *R. delavayi* plants used in this study. **(A)** Three-year-old potted, healthy young plants of *R. delavayi* that had not reached the reproductive growth stage. **(B)** Adult plants of *R. delavayi* were used in this study in the Kunming Jindian Scenic Area. **(C-G)**. Young plants under 9, 10, 11, 12, and 13 days drought stress, respectively, with severe leaf wilting at 11th, 12^th^, and 13th day of stress. **(H)** Fully recovered plants after rewatering from 13 days of drought stress.

This study utilized 3-year-old potted, healthy young plants of *R. delavayi* that had not reached reproductive growth stage as young plants (Figure 1A). The young plants were well-watered during the study and tissues: matured leaves (ML) and stem tips (STM) were sampled on the sampling dates presented in Table 1. On the other hand, the adult plants of *R. delavayi* were used in this study in the Kunming Jindian Scenic Area (Figure 1B), 1980 m above sea level, longitude and latitude of 102°43′ 5.99″E and 25°02′ 20.00″N, respectively, and tissues such as ML and flower bud (FB) were sampled at the dates given in Table 1.

**TABLE 1:** Sampling periods used for tissue collection during the study.

3-year-Old young plants (control)				Matured leaves from young drought-stressed plants			Adult plants		
Sampling date	Mature leaves	Stem tip		Number of day of drought stress	Code		Sampling date	Flower bud	Mature leaves
9/22/2017	ML-1	STM-1		9	B1-9		9/22/2017	FB-8	ML-8
10/8/2017	ML-2	STM-2		10	B1-10		10/8/2017	FB-9	ML-9
11/8/2017	ML-3	STM-3		11	B1-11		11/8/2017	NA*	ML-10
12/8/2017	ML-4	-		12	B1-12		12/8/2017	FB-11	ML-11
1/8/2018	ML-5	-		13	B1-13		1/8/2018	FB-12	ML-12
1/23/2018	ML-6	-		-	-		1/23/2018	FB-13	ML-13
2/8/2018	ML-7	-		-	-		2/8/2018	FB-14	ML-14
-	-	-		-	-		2/26/2018	FB-15	ML-15
* Not applicable									

To validate the expression of the selected candidate reference genes, some of the young plants of *R. delavayi* received no water for 9, 10, 11, 12, and 13 days during the study (Figures 1C–G, respectively). After the 13th day drought treatment, the plants were well-watered, leading to full recovery as evidenced in Figure 1H. MLs were sampled from the recovered plant at periods shown in Table 1. Specifically, tissue sampling was carried out sequentially simultaneously each day (10:30–11:00 a.m.), once per day for four consecutive days with three replicates.

### RNA Extraction and cDNA Synthesis

Total RNA was extracted following the manufacturer’s instructions with the RNAprep Pure Plant Kit (TianGen, Gen). The total RNA integrity was assessed *via* electrophoresis with 1% agarose gel. The quality and concentrations of RNA were measured by using a NanoDrop 2000 Spectrophotometer (Thermo Scientific Wilmington, DE, United States). First-strand cDNA was synthesized by reverse transcription of 300 ng total RNA with PrimeScript II First Strand cDNA Synthesis Kit Mix (Biotek, Beijing, China) with oligo (dT) primers in a final volume of 20 μL following the manufacturer’s protocols. All the cDNA samples were stored at −20°C for later use.

### Selection of Reference Genes and qRT-PCR

The full length sequences of eleven candidate housekeeping genes used in several previous studies in other crop species were used to blast *R. delavayi*’s whole-genome sequencing data released by Zhang et al. (2017) through nucleotide blast option (BlastN) using BioEdit software (Hall, 1999). Gene-specific primers for qRT-PCR analysis were designed using Primer Premier 6 standalone version (www.premierbiosoft.com), according to the sequences of the 11 candidate reference genes obtained from the *R. delavayi* genome (Table 2). The primers were synthesized by Tsingke Biotechnology Co., Ltd (Beijing, China).

**TABLE 2:** Details of primer pairs used in real-time fluorescence quantitative PCR for candidate genes and validation of genes in *R. delavayi*.

Gene symbol	Gene ID^a^	Genebank Accession no	Description	Primer sequences (5’—>3′)
*GAPDH*	*DUH011791.1*	MZ221074	Glyceraldehyde-3-phosphate dehydrogenase	F: TGT​TCG​TTA​TGG​GGG​TGA​ATG​AGA​A
				R: AGA​CCC​TCA​AGA​ATG​CCA​AAC​CTA​T
*Actin*	*DUH012597.1*	MZ221075	Actin	F: ATC​CAG​GCC​GTT​CTC​TCT​CTA​TA
				R: GTT​CGG​CCG​TGG​TAG​TGA​ACA
*EF1α*	*DUH018457.1*	MZ221076	Elongation factor 1-alpha	F: TGT​GCC​ATC​CTC​ATT​ATT​GAC​TCC
				R: ATG​GGA​TCT​TCT​CGG​GAT​TGT​ATC
*Tubulin-β-5*	*DUH015949.1*	MZ221077	Tubulin-β-5	F:TGCTGATGAGTGTATGGTTTTGGA
				R:AAATCAAATGGTTCAAATCTCCGA
*Ubiquitin*	*DUH018687.1*	MZ221078	Ubiquitin-40S ribosomal protein S27a	F: TCG​TGA​AAA​CCC​TAA​CGG​GCA
				R: CGG​AGG​ACG​AGG​TGG​AGG​GTG
*UEP*	*DUH018193.1*	MZ221079	Ubiquitin domain-containing protein	F:CCTCGCTGACTACAACATCCAGAA
				R:AGTAATGACGATCGAAGTGATTCGC
*UEC1*	*DUH004331.1*	MZ221080	Ubiquitin-conjugating enzyme E2-28	F:CAGGCGGAGTTTTTCTTGTTACCA
				R:GGGCTCCACTGCTCCTTTAAGATA
*UEC2*	*DUH006428.2*	MZ221081	Ubiquitin-conjugating enzyme E2-32	F:GGGTGAAGAGGATTCTACAGGAGG
				R:GACCATTTGGCGTCAACAACATAA
*TIP41*	*DUH005805.1*	MZ221082	TIP41-like family protein	F:AGAAGCAACATCTGAAAAGGGCAA
				R:CATCAACTCTAAGCCAGAAACGCA
*Tubulin beta*	*DUH031839.1*	MZ221083	Tubulin beta-2 chain	F:CTCACTACTCCCAGTTTTGGCGAT
				R:GAGCGGAGCAAAACCAACCATAA
*TATA*	*DUH029306.1*	MZ221084	TATA-box- binding protein	F:TCCTGCGATGTAAAATTTCCTATCC
				R:CCGACACAAAGATGAGAAGCACAA
*NCED1*	*DUH017412.1*		9-*cis*-epoxycarotenoid dioxygenase	F:AAATCACACCCAACGGGGACTT
				R:CATCATTGTCGCCTCATTCAGTG

a Gene identification number in *R. delavayi*’s genome.

To detect the specificity and amplification efficiency of each pair of primers developed, we conducted RT-PCR in a 20-μl system using a Mastercycler nexus GSXI PCR apparatus (Eppendorf AG, Hamburg, Germany) following the manufacturer’s instructions. Thus, 60 ng of the synthesized cDNA, 2 μl rTaq buffer (TakaRa, Dalian, China), 0.4 μM of two primer pairs equally and thoroughly mixed, 200 μM each dNTP, and 1 U rTaq (TaKaRa, Dalian, China) were used. The amplification program and product evaluation were conducted following the conditions outlined by Jin et al. (2019). The dissociation curve was generated by melting the amplicons from 60–95°C.

All qRT-PCR experiments were performed in three biological replicates and three technical replicates. A no template control and reverse transcription negative control were also performed. The relative gene expression levels were computed using the 2^-∆∆Ct^ method (Livak and Schmittgen, 2001).

### Analysis of the Stabilities of Selected Genes

Five tools– Delta C_t_ method (Silver et al., 2006), BestKeeper (Pfaffl et al., 2004), geNorm (Vandesompele et al., 2002), Normfinder (Andersen et al., 2004), and RefFinder (Xie et al., 2012) were used to compute the stabilities of the individual genes. Briefly, the Delta CT method was used to select the optimal reference gene by comparing the relative expression of “pairs of genes” within each sample (Silver et al., 2006). BestKeeper works with ranking of candidate reference genes based on standard deviations and coefficients of variation (CVs) (Pfaffl et al., 2004). geNorm was used to compute the normalization factor to determine the optimal number of reference genes following the pairwise variation (V) between different candidate genes, thus V_n_/V_n+1_. When the ratio is less than 0.15, it implies that the number of internal reference gene combinations in this group can maintain the accurate normalization of the data to some extent (Vandesompele et al., 2002). The Normfinder algorithm is a model-based approach which ranks the candidate reference genes by the stability value, of which the gene with the lowest value represents the most stable reference gene (Andersen et al., 2004). Last, the RefFinder (https://www.heartcure.com.au/reffinder/?type=reference accessed on 22/12/2021) assigns an appropriate weight of the four methods elaborated previously to a single gene and computes the geometric average of their weights for the overall final ranking (Xie et al., 2012).

### Validation of Identified Reference Genes

The *NCED1* copy in the *R. delavayi* genome (*DUH017412.1*), which catalyzes oxidative cleavage of 9-*cis*-epoxycarotenoids neoxanthin and violaxanthin to xanthoxin, is a key step in the biosynthesis of abscisic acid (ABA) in higher plants (Changan et al., 2018). The most and least stable reference gene and the combination of the two–three best reference genes were used to normalize the expression profile of *DUH017412.1* in the tissues sampled at a young stage with ML. The relative expression was conducted with the comparative 2^-∆∆Ct^ method (Livak and Schmittgen, 2001).

### Statistical Analysis and Visualization

Data obtained via qRT-PCR were subjected to analysis of variance (ANOVA), and *post hoc* mean separation was carried out by Duncan’s Multiple Range Test at *p < 0.05* using GenStat software (Version 12; https://www.vsni.co.uk/)*.* Means were visualized *via* GraphPadPrism (Version 7; https://www.graphpad.com/). Transcriptome data of the candidate reference genes and *NCED1* were log2 transformed and heat-mapped with the *pheatmap* package in R (Kolde, 2012).

## Results

### Primer Specificity and Amplification Efficiency of the Candidate Reference Genes

We blasted the genome of *R. delavayi* earlier published by Zhang et al. (2017) with 11 candidate reference genes listed in Tables 2–3. The melting curves of the 11 candidate reference genes showed that all primer pairs designed present a single peak (Supplementary Figure S1), which validates the specificity of primers. The amplicon lengths of the 11 candidate reference genes ranged from 103 bp in *Tubulin-β-5* to 257 bp in *TIP41* (Table 3, Supplementary Figure S2). On the other hand, the minimum (90.30%) and maximum (105.60%) amplification efficiency was observed in *UEC1* and *Actin*, respectively (Table 3). Strong correlation coefficients (R = 1) were recorded for the eleven candidate reference genes. The linear equation predicted based on Ct for the primers’ specificity is shown in Table 3. From this background, the primers were used for further experiments aimed at screening the 11 candidate reference genes along with one reference gene (*NCED1*) for their stabilities.

**TABLE 3:** Primers used in this study and their amplification efficiency and characteristics for eleven candidate reference genes in *R. delavayi*.

Gene symbol	Tm (°C)^a^	Al (bp)^b^	E (%)^c^	R^d^	Equa^e^
*GAPDH*	58	133	103.10	0.997	Y = -3.25x+17.54
*Actin*	58	211	105.60	0.992	Y = -3.10x-21.73
*EF1α*	58	238	100.10	0.995	Y = -3.27x+18.58
*Tubulin-β-5*	58	103	90.70	0.993	Y = -3.14x+25.22
*Ubiquitin*	58	205	99.80	0.995	Y = -3.31x+17.41
*UEP*	58	257	102.10	0.991	Y = -4.44x+15.85
*UEC1*	58	148	90.30	0.994	Y = -4.35x+19.43
*UEC2*	58	219	99.00	0.996	y = -4.75x+18.79
*TIP41*	58	257	97.40	0.993	Y = -3.68x+21.86
*Tubulin-β*	58	171	93.10	0.997	Y = -3.37x+22.95
*TATA*	58	142	95.80	0.998	Y = -3.41x+24.20

a Annealing temperature.

b Amplicon length.

c Amplification efficiency.

d Correlation coefficient.

e Linear equation.

### Expression Profiles of the Candidate Reference Genes in Different Tissues in Young and Adult Plants Under Control and Drought Stress Conditions

**FIGURE 2:**
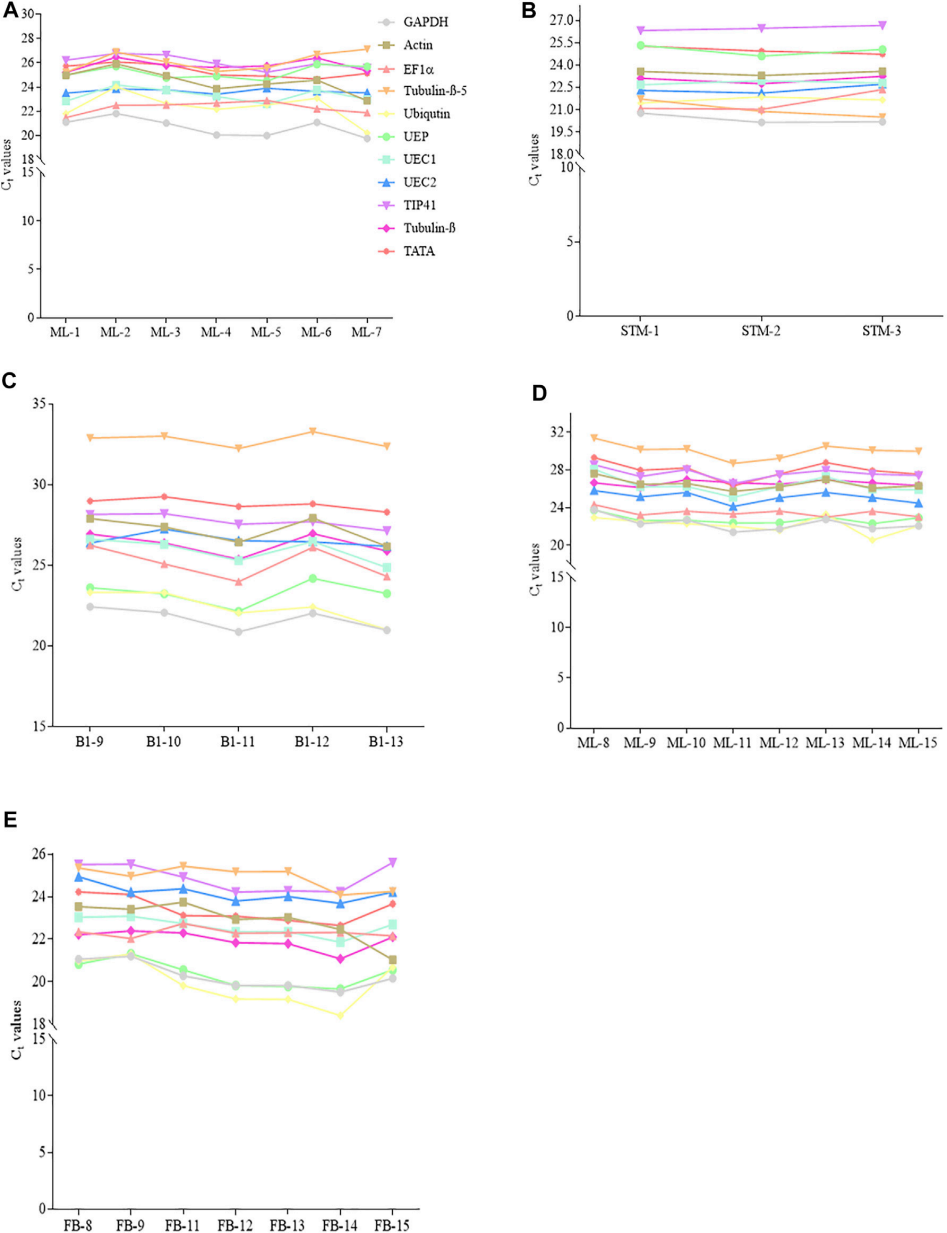
Threshold cycle (C_t_) of the 11 candidate reference genes in young and adult *R. delavayi*. **(A)** Young matured leaves (ML) (sampled at 9/22/2017, 10/8/2017, 11/8/2017, 12/8/2017, 1/8/2018, 1/23/2018, and 2/8/2018 denoted ML1, ML2, ML3 ML4, ML5, ML6, and ML7, respectively). **(B)** Stem tips sampled from young plant (at 9/22/2017, 10/8/2017, and 11/8/2017 denoted STM-1, STM-2, and STM-3, respectively). **(C)** Drought stress samples (sampled at 9, 10, 11, 12, and 13 days after drought stress (DS) condition denoted B1-9, B1-10, B1-11, B1-12, and B1-13, respectively). **(D)** Adult ML (sampled at 9/22/2017, 10/8/2017, 12/8/2017, 1/8/2018, 1/23/2018, 2/8/2018, and 2/26/2018 denoted ML8, ML9, ML11, ML12, ML13, ML14, and ML15, respectively). **(E)** Flower bud (FB) (sampled at 9/22/2017, 10/8/2017, 12/8/2017, 1/8/2018, 1/23/2018, 2/8/2018, and 2/26/2018 denoted FB8, FB9, FB11, FB12, FB13, FB14, and FB15, respectively).

**FIGURE 3:**
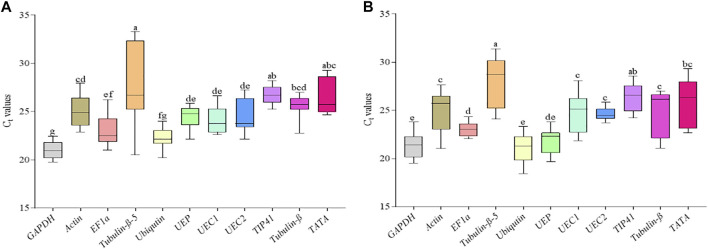
Threshold cycle (C_t_) of the 11 candidate reference genes in the aboveground parts of young and matured *R. delavayi* plant. **(A)** Young plant (matured leaves (ML) + stem tips (STM) + under drought stress (DS) condition. **(B)** Matured plant (ML + STM + flower buds (FB)). Average of the 2 years (2017 and 2018 sampling periods). Lower and upper lines on the box represent the minimum and maximum C_t_, respectively. The lower and upper parts of the box represent 25 and 75% percentile, respectively, and the line in the middle of the box represents mean Cq for each gene. Boxes with a common alphabet indicate no significant difference (*p* > 0.05), while those with no common alphabet indicate significant difference (*p* < 0.05) with *Post hoc* mean separation by Duncan’s Multiple Range Test.

The transcript abundance of the eleven candidate reference genes in matured leaves (ML) and stem tips (STM) under control and stress conditions sampled from young *R. delavayi* and ML and flower buds (FB) from adult *R. delavayi* were determined by C_t_ values from qRT-PCR experiments (Figures 2A–E). The results revealed that the candidate reference genes showed a wide range of transcription level across all the test samples under control and drought stress conditions at different sampling times (Figures 3A–E). The ML sample from young *R. delavayi* had transcript abundance ranging from 21.09 in *GAPDH* to 27.12 in *Tubulin-β-5* (Figure 2A). Consistently, *GAPDH* had the least C_t_ values across the seven sampling times in ML sampled from young *R. delavayi*, while *TIP41* had the highest C_t_ values from ML1–ML4; the remaining three sampling times (ML5–ML7) had *Tubulin-β-5* recording the highest C_t_ values (Figure 2A). In the case of the STM, *GAPDH* and *TIP41* had the least (20.16–20.79) and maximum (26.33–26.68) C_t_ values, respectively (Figure 2B).

Under drought stress condition, *GAPDH* had the least C_t_ values (20.88–22.44), while *Tubulin-β-5* exhibited the highest C_t_ values (32.24–33.29) in the ML sampled from young *R. delavayi* (Figure 2C). This suggests that environmental factors such as drought could alter the transcript abundance of candidate reference genes, indicating the relevance of our study.

In addition to the previously mentioned data, we sampled ML and FB from adult *R. delavayi* to evaluate the expression of the eleven candidate reference genes *via* C_t_ values. With ML from adult plants, *Tubulin-β-5* had the highest C_t_ values (28.71–31.40), while either *Ubiquitin* and *GAPDH or UEP* had the least C_t_ values (20.58–23.77) across the eight sampling times (Figure 2D). A similar trend was observed in FB as in the ML, but the highest C_t_ values were observed with either *Tubulin-β-5* or *TIP41* (Figure 2E).

We further pooled the C_t_ values recorded previously and treated each as a replicate for each of the candidate reference genes and subjected to ANOVA. It was observed that significant variation existed among the tissues from young (plus under drought stress) and adult *R. delavayi* (Figures 3A,B). The C_t_ values of *Tubulin-β-5* had transcript abundance in the young *R. delavayi* under control and drought stress with wide variation in the range and mean ± standard deviation (20.52–33.29 and 27.32 ± 4.48), which statistically differed from *TIP41* (25.23–28.20 and 26.70 ± 0.90) and *TATA* (24.67–29.26 and 26.96 ± 1.80), while the mean C_t_ values recorded by *GAPDH* (20.96 ± 0.84) and *Ubiquitin* (22.53 ± 1.06) were statistically similar (Figure 3A). With exception of *TATA*, *Tubulin-β-5* and *TIP41* and *GAPDH* (20.96 ± 0.84) and *Ubiquitin* in adult *R. delavayi* followed similar statistical order as observed in young *R. delavayi* (Figure 3B).

### *In Silico* Expression Profiles of the Candidate Reference Genes in Dormant Buds and Drought Condition

**FIGURE 4:**
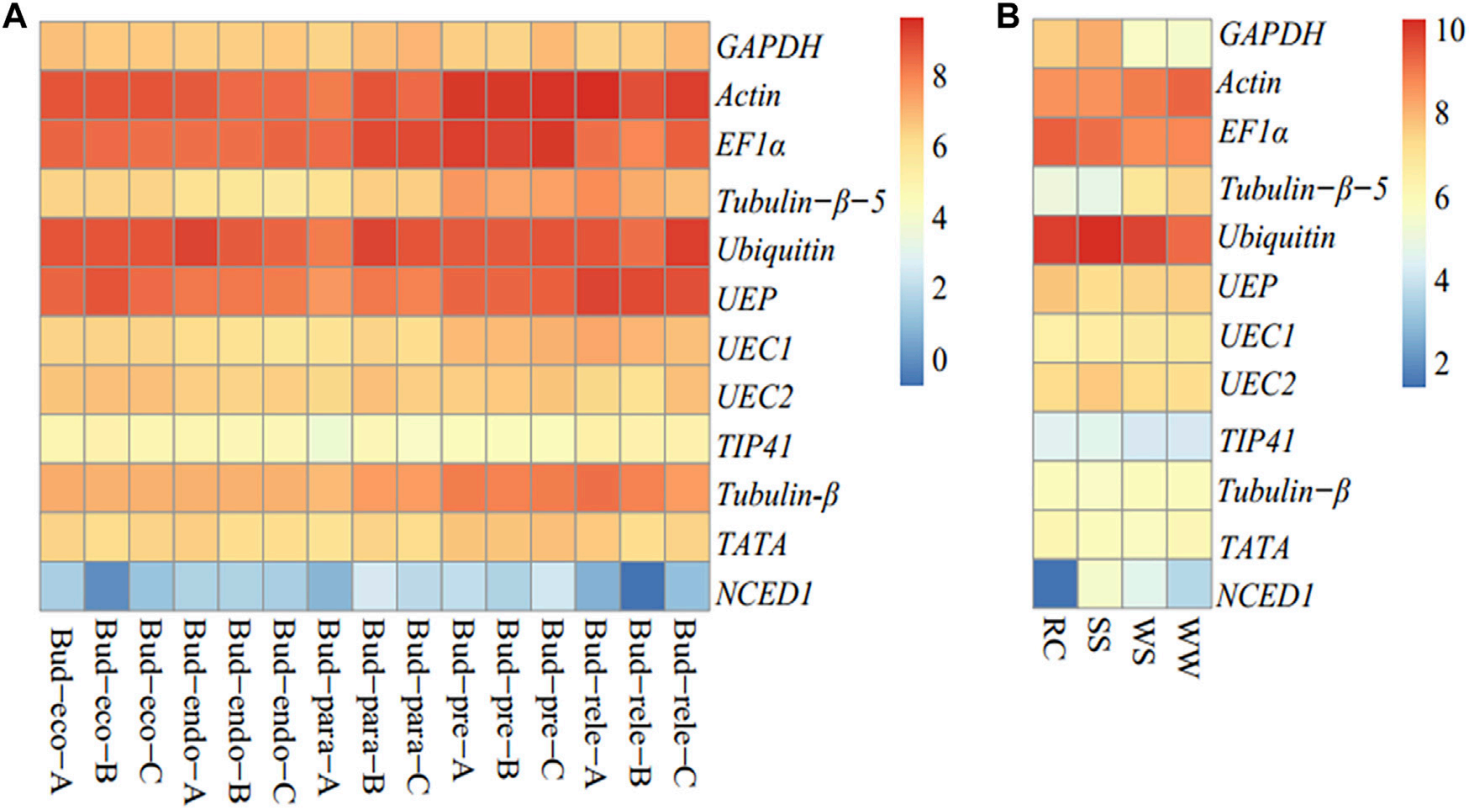
Gene (eleven candidate reference genes and *NCDE1*) expression based fragments per kilobase of transcript per million mapped reads (log2 transformation) in *R. delavayi*. **(A)** Dormant buds from the study with accession SRA = PRJNA476831 (available at https://www.ncbi.nlm.nih.gov/bioproject/476831), pre-dormancy (Bud-pre, 07/20/2015), para-dormancy (Bud-para, 10/29/2015), endo-dormancy (Bud-endo, 12/02/2015), eco-dormancy (Bud-eco, 12/27/2015), and dormancy-release (Bud-rele, 01/14/2016)). **(B)** Drought stress condition from the study of Cai et al. (2019) (SRA = PRJNA503304) (four experimental scenarios were designed: normal irrigation (WW), stopping irrigation for 5 days (WS), stopping irrigation for 9 days (SS), and re-watering for 6 h (REC) after 10 days of drought. Rewatering was only carried out on 10 plants. The other five plants were continued under no irrigation for subsequent electron microscopic observation. All the measurements were carried out at the end of each timepoint).

In order to give credence to the transcript abundance assessed by C_t_ values for the eleven candidate reference genes and one reference gene (*NCED1*), we retrieved gene expression based on fragments per kilobase of transcript per million (FPKM) mapped reads from two project data available on the NCBI with accession numbers: PRJNA476831 (dormant bud) and PRJNA503304 (drought) (Cai et al., 2019). The FPKM was log2-transformed and heat-mapped with the *Pheatmap* package in R (Kolde, 2012). It was observed that *Actin, EF1α*, *Ubiquitin*, and *UEP* consistently exhibited the highest FPKM across the dormant buds, while the least FPKM was observed with *NCED1* (Figure 4A).

Under drought conditions, A*ctin*, *EF1α*, and *Ubiquitin* persistently had the highest transcript abundances under the five conditions evaluated under drought treatments: normal irrigation (WW), stopping irrigation for 5 days (WS), stopping irrigation for 9 days (SS), stopping irrigation for 10 days, and re-watering for 6 h (REC) after 10 days of drought, whereas the reference gene (*NCED1*) had the transcript abundance in the order of SS > WS > WW > RC. This trend gives credence to our use of *NCED1* as the reference gene to validate our results under drought.

### Stability Analyses of Candidate Reference Genes in *R. delavayi*

Under drought condition, the best five stable candidate reference genes followed the order *GAPDH* > *UEC1* > *Actin* > *Tubulin-β > Tubulin-β-5* and were predicted by the C_t_ method for ML (Table 4), whereas the BestKeeper method predicted *TATA, UEC2, TIP41*, and *Tubulin-β-5* as the four most stable candidate reference genes. The geNorm predicted *Actin* together with *UEC1* genes as the most stable followed by *GAPDH* > *UEC1* > *Actin* > *Tubulin-β* (Table 4). The *GAPDH* followed by *UEC1*, *Tubulin-β > Tubulin-β-5*, and *Actin* were predicted as the best five stable candidate reference genes by Normfinder. By integrating the results from C_t_, BestKeeper, geNorm, and Normfinder, the RefFinder predicted *GAPDH* > *UEC1* > *Actin* > *Tubulin-β-5* as the four most stable candidate reference genes under drought stress condition with ML as the tissue. Consistently among the five tools used for stability analysis of the candidate reference genes, *Ubiquitin* was among the last two most stable genes (Table 4).

**TABLE 4:** Stability ranking of 11 candidate reference genes with matured leaf sampled from a 3-year-old young *R. delavayi* under drought stress.

Stability ranking	C_t_			BestKeeper			geNorm			Normfinder			RefFinder	
	Gene	STDEV		Gene	STDEV		Gene	Stability value		Gene	Stability value		Gene	Geomean
1st	*GAPDH*	0.42		*TATA*	0.27		*Actin + UEC1*	0.15		*GAPDH*	0.11		*GAPDH*	2.14
2nd	*UEC1*	0.45		*UEC2*	0.28		*GAPDH*	0.22		*UEC1*	0.21		*UEC1*	2.38
3rd	*Actin*	0.47		*TIP41*	0.34		*Tubulin-β*	0.27		*Tubulin-β-5*	0.24		*Actin*	3.41
4th	*Tubulin-β*	0.47		*Tubulin-β-5*	0.37		*Tubulin-β-5*	0.33		*Tubulin-β*	0.27		*Tubulin-β-5*	4.16
5th	*Tubulin-β-5*	0.47		*UEP*	0.49		*TIP41*	0.37		*Actin*	0.27		*TATA*	4.30
6th	*TIP41*	0.49		*Tubulin-β*	0.55		*TATA*	0.39		*TIP41*	0.32		*Tubulin-β*	4.43
7th	*TATA*	0.51		*GAPDH*	0.60		*EF1α*	0.44		*TATA*	0.37		*TIP41*	5.05
8th	*EF1α*	0.65		*UEC1*	0.67		*UEP*	0.47		*UEP*	0.56		*UEC2*	6.85
9th	*UEP*	0.65		*Actin*	0.69		*UEC2*	0.51		*EF1α*	0.57		*UEP*	7.55
10th	*UEC2*	0.68		*Ubiquitin*	0.71		*Ubiquitin*	0.54		*Ubiquitin*	0.60		*EF1α*	8.92
11th	*Ubiquitin*	0.69		*EF1α*	0.82		*-*	-		*UEC2*	0.61		*Ubiquitin*	10.49

The use of young *R. delavayi* with both ML and STM under control and ML from drought stress condition was considered, and the C_t_ method predicted *UEC1*, *Actin*, *UEC2*, and *GAPDH* as the four most stable candidate reference genes (Table 5). In addition, *GAPDH*, *TIP41*, *Ubiquitin*, and *UEP* were predicted as the best candidate reference genes by the BestKeeper method, while the geNorm method predicted *UEC2*+*TATA* as the best candidate reference gene followed by *UEC1* > *Actin* > *EF1α*. Normfinder predicted that *UEC1* > *Actin* > *UEC1*>*EF1α* as the four stable genes. The results from RefFinder prediction for the best four candidate reference genes followed similarly with the C_t_ method with minor rearrangement. From the five models, *Tubulin-β-5* was ranked the least stable which is consistent with Figure 4A, suggesting that the wider the range of C_*t*_ values, the lesser the stability will be.

**TABLE 5:** Stability ranking of 11 candidate reference genes with tissues (matured leaf and stem) and under drought conditions (matured leaf) from young *R. delavayi*.

Stability ranking	C_t_		BestKeeper		geNorm		Normfinder		RefFinder	
	Gene	STDEV	Gene	STDEV	Gene	Stability value	Gene	Stability value	Gene	Geomean
1st	*UEC1*	1.22	*GAPDH*	0.65	*UEC2+TATA*	0.57	*UEC1*	0.28	*UEC1*	2.06
2nd	*Actin*	1.30	*TIP41*	0.71	*UEC1*	0.63	*Actin*	0.33	*UEC2*	3.00
3rd	*UEC2*	1.35	*Ubiquitin*	0.76	*Actin*	0.65	*UEC2*	0.45	*Actin*	3.36
4th	*GAPDH*	1.36	*UEP*	0.83	*EF1α*	0.68	*EF1α*	0.55	*GAPDH*	3.60
5th	*EF1α*	1.38	*Tubulin-β*	0.99	*TIP41*	0.80	*TATA*	0.69	*TATA*	4.33
6th	*TIP41*	1.38	*UEC1*	1.23	*GAPDH*	0.87	*GAPDH*	0.71	*TIP41*	4.74
7th	*TATA*	1.42	*EF1α*	1.37	*Tubulin-β*	0.96	*TIP41*	0.73	*EF1α*	5.14
8th	*Tubulin-β*	1.53	*Actin*	1.42	*Ubiquitin*	1.05	*Tubulin-β*	0.80	*Ubiquitin*	6.84
9th	*Ubiquitin*	1.67	*UEC2*	1.46	*UEP*	1.27	*Ubiquitin*	1.26	*Tubulin-β*	7.11
10th	*UEP*	2.47	*TATA*	1.59	*Tubulin-β-5*	1.70	*UEP*	2.33	*UEP*	7.95
11th	*Tubulin-β-5*	3.63	*Tubulin-β-5*	3.63	*-*	-	*Tubulin-β-5*	3.56	*Tubulin-β-5*	11.00

*TIP41* was predicted as the most stable candidate reference gene in the tissues of adult *R. delavayi* (ML + FB) followed *UEP*, *UEC1*, and *GAPDH* by the C_t_ method (Table 6). Conversely, the BestKeeper method classified *EF1α*, *UCE2*, *GAPDH*, and *UEP* as the top four stable candidate reference genes, but in the case of geNorm, *GAPDH + UEP*, *TIP41*, *Ubiquitin*, and *UEC2* were considered the best four candidate reference genes. The first four best candidate reference genes predicted by Normfinder followed a similar trend as that of the C_t_ method (Table 5). A comprehensive analysis by RefFinder revealed *UEP*, *TIP41*, *GAPDH*, and *EF1α* as the best candidate reference genes (Table 6).

**TABLE 6:** Stability ranking of 11 candidate reference genes with tissues (matured leaf and flower bud) sampled from adult *R. delavayi*.

Stability ranking	C_t_		BestKeeper		geNorm		Normfinder		RefFinder	
	Gene	STDEV	Gene	STDEV	Gene	Stability value	Gene	Stability value	Gene	Geomean
1st	*TIP41*	0.87	*EF1α*	0.58	*GAPDH + UEP*	0.35	*TIP41*	0.24	*UEP*	2.00
2nd	*UEP*	0.88	*UCE2*	0.61	*TIP41*	0.41	*UEP*	0.36	*TIP41*	2.06
3rd	*UEC1*	0.93	*GAPDH*	1.02	*Ubiquitin*	0.51	*UEC1*	0.46	*GAPDH*	2.63
4th	*GAPDH*	0.94	*UEP*	1.20	*UEC2*	0.67	*GAPDH*	0.54	*EF1α*	4.70
5th	*Actin*	1.04	*Ubiquitin*	1.21	*EF1α*	0.73	*Actin*	0.63	*UEC1*	4.74
6th	*Ubiquitin*	1.10	*TIP41*	1.36	*UEC1*	0.84	*Ubiquitin*	0.79	*Ubiquitin*	5.18
7th	*TATA*	1.14	*Actin*	1.81	*Actin*	0.90	*TATA*	0.92	*UEC2*	5.62
8th	*Tubulin-β*	1.21	*UEC1*	1.89	*TATA*	0.99	*Tubulin-β*	0.99	*Actin*	6.12
9th	*EF1α*	1.32	*TATA*	2.28	*Tubulin-β*	1.04	*EF1α*	1.17	*TATA*	7.94
10th	*UEC2*	1.32	*Tubulin-β*	2.32	*Tubulin-β-5*	1.10	*UEC2*	1.19	*Tubulin-β*	8.94
11th	*Tubulin-β-5*	1.37	*Tubulin-β-5*	2.56	*-*	-	*Tubulin-β-5*	1.25	*Tubulin-β-5*	11.00

qRT-PCR is usually conducted on different tissues at varied developmental stages and conditions (control and stressed); hence, we pooled the C_t_ values together to predict their stability in both young (control-ML + STM and under DS) and adult (ML + FB) *R. delavayi* (Table 7). The C_t_ method predicted *TIP41* as the most stable candidate reference gene followed by *Actin*, *UEC1*, and *GAPDH*. Equally, *GAPDH*, *EF1α*, *TIP41*, and *UEC2* were predicted as the best four candidate reference genes by the BestKeeper method (Table 6). geNorm recommended *GAPDH + TIP41* as the best gene pair followed by *UEC1*, *Actin*, and *EF1α*, whereas Normfinder predicted *Actin*, *TIP41*, *UEC1*, and *GAPDH* as the best four candidate reference genes (Table 7). The five statistical algorithms ranked *Tubulin-β-5* as the most unstable candidate reference gene, suggesting that the higher the average C_t_ values, the poorer the stability will be.

**TABLE 7:** Stability ranking of 11 candidate reference genes with tissues from young (matured leaf and stem) and under drought (matured leaf) and adult (matured leaf and flower bud) of *R. delavayi*.

Stability ranking	C_t_		BestKeeper		geNorm		Normfinder		RefFinder	
	Gene	STDEV	Gene	STDEV	Gene	Stability value	Gene	Stability value	Gene	Geomean
1st	*TIP41*	1.26	*GAPDH*	0.87	*GAPDH + TIP41*	0.64	*Actin*	0.47	*TIP41*	1.57
2nd	*Actin*	1.29	*EF1α*	0.96	*UEC1*	0.80	*TIP41*	0.51	*GAPDH*	2.00
3rd	*UEC1*	1.31	*TIP41*	1.03	*Actin*	0.86	*UEC1*	0.59	*Actin*	2.74
4th	*GAPDH*	1.36	*UEC2*	1.05	*EF1α*	0.96	*GAPDH*	0.76	*UEC1*	3.83
5th	*TATA*	1.43	*Ubiquitin*	1.06	*UEC2*	0.99	*TATA*	0.78	*EF1α*	4.36
6th	*EF1α*	1.46	*UEP*	1.55	*TATA*	1.03	*EF1α*	0.89	*UEC2*	6.06
7th	*UEC2*	1.49	*Actin*	1.59	*Ubiquitin*	1.11	*Tubulin-β*	0.92	*TATA*	6.47
8th	*Tubulin-β*	1.52	*UEC1*	1.61	*Tubulin-β*	1.17	*UEC2*	0.97	*Ubiquitin*	7.55
9th	*Ubiquitin*	1.55	*Tubulin-β*	1.76	*UEP*	1.35	*Ubiquitin*	1.09	*Tubulin-β*	8.21
10th	*UEP*	2.31	*TATA*	1.88	*Tubulin-β-5*	1.62	*UEP*	2.13	*UEP*	8.80
11th	*Tubulin-β-5*	2.79	*Tubulin-β-5*	3.13	*-*	-	*Tubulin-β-5*	2.65	*Tubulin-β-5*	11.00

### Validation of the Selected Candidate Reference Genes Under Drought Stress Condition

**FIGURE 5:**
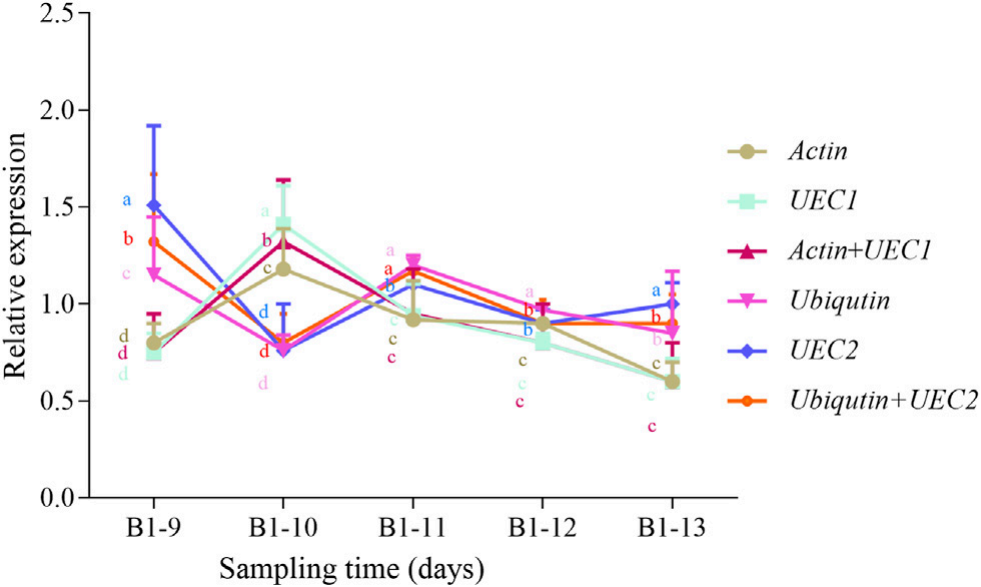
Relative expression of selected genes: most stable (*Actin* + *UEC1*) and most unstable (*UEC2* + *TIP41*) relative to the reference gene (*NCED1*) under drought stress condition of matured leaves of young *R. delavayi* plant. Sampling times consisted of 9, 10, 11, 12, and 13 days of drought stress (no watering) condition denoted B1-9, B1-10, B1-11, B1-12, and B1-13, respectively. The error bars represent standard deviations computed on three replications. Different letters of the same colors of the lines represent significant difference (*p < 0.05*) at each time point based on Duncan’s Multiple Range Test.

In order to evaluate the reliability of candidate reference genes, we selected two most stable (*Actin* and *UEC1*) and most unstable (*UEC2* and *Ubiquitin*) either individually or in combination from the geNorm model and normalized their Ct values with *NCED1* to obtain their relative expression level in matured leaf samples at five sampling times from young *R. delavayi* (Figure 5). Mostly, the expression patterns of most stable and unstable genes were similar with minor discrepancies. For example, at B1-9 sampling time, either one or a combination of the most unstable candidate reference genes (*UEC2* and *Ubiquitin*) had significantly different relative expressions, of which either was statistically higher and different from the two most stable (*Actin* and *UEC1*) (Figure 5). However, contrary trends were observed at B1-10. As evidenced by standard deviation at each sampling time, *UEC2* and *Ubiquitin*, either individually or in combination, were unstable when used, confirming the results of the geNorm model under drought condition (Table 4). This result highlights the need to select a suitable reference gene for gene expression studies.

## Discussion

Though the draft genome of *R. delavayi* was announced in 2017 by Zhang et al. (2017), there has been no report on screening reference genes for qRT-PCR experiments, unlike other crops such as soybean (Gao et al., 2017), barley (Janská et al., 2013), flax (Huis et al., 2010), bamboo (Fan et al., 2013), rice (Almas and Kamrodi, 2018), and tomato (Cheng et al., 2017). Gene expression analysis by qRT-PCR in plants of economic, nutritional, and health importance has gained increasing attention in recent years (Vandesompele et al., 2002; Almas and Kamrodi, 2018; Ahiakpa et al., 2021; Sun et al., 2021; Xiao et al., 2021) as a result of its high sensitivity, quantitative accuracy, low cost, and high throughput (Bustin, 2002; Radonić et al., 2004). One of the determinants of successful and meaningful qRT-PCR operation is the selection and reliability of reference genes which function to normalize the qRT-PCR experiment to ensure that results are both statistically significant and biologically meaningful (González-Aguilera et al., 2016).

To screen and select a number of candidate genes for functional validation in *R. delavayi via* gene overexpression, virus-induced gene silencing (VIGS) and clustered regularly interspaced short palindromic repeats (CRISPR)/CRISPR-associated protein (Cas) (CRSIPR/Cas) technology (Zhang et al., 2022) from our transcriptomics dataset were carried out*,* and our research group attempted to validate the expression profile of RNA-sequencing (RNA-seq) data by qRT-PCR; however, the challenge of the reference gene was eminent; hence, we decided to screen 11 known reference genes reported in other crops (Fan et al., 2013; Gao et al., 2017; Yang et al., 2019; Jin et al., 2019). First, we blasted the eleven known reference genes with the draft genome of *R. delavayi* and designed primer pairs for each of the genes (Table 2). One of the requirements for primer pairs for qRT-PCR is the specificity of the primers for a specific gene (David et al., 2017; Bustin and Huggett, 2017). Second, we performed primer-specificity experiments for the 11 candidate reference genes *via* melting curve and analyzed amplification efficiencies and correlation coefficients which had single peak (Supplementary Figure S1), 90.30–105.60% and 0.993–0.998 (Table 3), respectively. These primer specificity results revealed that all 11 pairs of primers meet the criteria for qRT-PCR experiments (Gao et al., 2017).

We subsequently screened the 11 candidate reference genes in matured leaves, stem tips, and flower buds at two developmental stages (young and adult) (Figures 2A,B, d–e). It was observed that the 11 candidate reference genes have varied expression values (C_t_) in different tissues and developmental stages. For instance, GAPDH was among the genes with least transcript abundance in all the tissues at the two different developmental stages (Figures 2A,B, d–e; Figures 3A,B). This according to Gao et al. (2017) suggests that the cDNA sample with more abundance reaches the threshold at lower C_t_ value, which leads to a higher gene expression level. The wide variation in C_t_ values is consistent with studies in *Camellia sinensis* and *Brassica napus.* Thus, the expression of the housekeeping genes varies considerably under different conditions and in different tissues (Hao et al., 2014; Yang et al., 2014).

*R. delavayi* is well known as an alpine evergreen ornamental plant, in which water shortage impedes its growth and development in urban gardens (Cai et al., 2019). With this in mind, our research group has initiated studies to unravel the molecular mechanism underlying drought tolerance/susceptibility in *R. delavayi* with the aim of developing more drought-resilient genotypes. Therefore, the eleven candidate reference genes were screened for their stability under drought stress. *GAPDH* is a multifunctional enzyme that plays an important role in abiotic stress including drought and plant development (Zhang et al., 2019). Consistently, across the five sampling times (B1–9 to B1–13), *GAPDH* had the least C_t_ value suggesting its highest gene expression among the eleven candidate reference genes. The study of Zhang et al. (2019) explained that overexpression of *GAPDH* encoded the gene *TaGAPC1* to enhance drought tolerance in transgenic *Arabidopsis*; therefore, we speculate that *GAPDH* (*DUH011791.1*) could be the candidate gene for drought tolerance in *R. delavayi*, as it also showed higher transcript abundance under drought stress (stopping irrigation for 9 days, SS) in the RNA-seq of Cai et al. (2019) (Figure 4B). A similar observation was made in maize and *Arabidopsis* (Russell and Sachs, 1992; Yang et al., 1993) under polyethylene glycol, hydrogen peroxide, and abscisic acid stress.

It is established that the ideal reference gene should exhibit a consistent expression level across all tested tissues or conditions (Bustin, 2002; Udvardi et al., 2008). To establish stability in the expression of the eleven candidate reference genes, we applied five statistical algorithms, of which RefFinder is a user-friendly web-based comprehensive tool developed for evaluating and screening reference genes from extensive experimental datasets (Xie et al., 2012) and integrates the four other computational programs (Delta Ct method (Silver et al., 2006)– BestKeeper (Pfaffl et al., 2004), geNorm (Vandesompele et al., 2002), and Normfinder (Andersen et al., 2004)). The ranking values of these programs indicate the most stable (least value), moderately stable (average), and least stable (highest value). Under drought condition with matured leaves as tissue, the five programs largely predicted *GAPDH* as the most stable reference gene together with *UEC1* and *Actin*, while the least was *Ubiquitin* (Table 4). These corroborate findings by Nicot et al. (2005), Garg et al. (2010), Li et al. (2012), and Müller et al. (2015). The essential roles these genes play in basic cellular progress may have accounted for their stable expression. However, Mao et al. (2021) reported that *GAPDH* had the least stability in pineapple (*Ananas comosus* var bracteatus) in response to hormone stimuli. The further validation of our results with two of the most stable candidate reference genes (*UEC1* and *Actin*) and two least stable genes (*UEC2* and *TIP41*), which were normalized by one well-known gene under drought, showed that *NCDE1* was consistent with our stability analysis (Table 4; Figure 5). This indicates that RNA-seq data from other projects provide clues on the expression of genes in tissues and conditions for their inclusion in qRT-PCR experiments. However, there is a need to conduct optimization experiments to confirm and select ideal reference genes for qRT-PCR.

On the contrary, RefFinder ranked *UEC1*, *UEC2*, *Actin*, and *GAPDH* as the topmost stable candidate reference genes at the young stage of *R. delavayi* with matured leaves and stem tips under control and drought stress conditions (matured leaves) and *Tubulin-β-5* as the least stable (Table 5). This indicates that no reference gene is adequate in all experimental settings and tissues and in all stages of development (Mao et al., 2021), which suggests that reference genes should be thoroughly evaluated prior to specific experimental conditions and use (Cheng et al., 2017). The ranking of *Tubulin-β-5* partly corroborates this finding, while *GAPDH* contrasts with the findings of Li et al. (2020).

*TIP41* has been considered a stable internal control gene for *Caragana intermedia* (Zhu et al., 2013) and in tall fescus (*Festuca arundinacea* Schreb) under several abiotic stresses such as salinity, drought, cold, and heat (Yang et al., 2015). This gene (*TIP41*) together with *GAPDH* was stably ranked among the topmost four potential reference genes by the five statistical models (Vandesompele et al., 2002; Andersen et al., 2004; Pfaffl et al., 2004; Silver et al., 2006; Xie et al., 2012) (Table 6-7) with matured leaves and flower buds and young (+drought stress) and adult combined from *R. delavayi.* Thus, either *TIP41* or *GAPDH*/their combination could be exploited for qRT-PCR experiments in *R. delavayi*. However, the criteria for the ranking among potential reference genes largely remain unknown; whether the differences are caused by the genes themselves or the difference caused by the specific amplified fragments that affect the amplification experiment warrants further investigations. In addition, future studies should include the use of several tissues such as root from drought and other abiotic stresses conditions to study the stabilities of the eleven and other novel candidate reference genes.

## Conclusion

The present study represents the first systematic identification and screening of potential reference genes as internal controls for normalization of qRT-PCR results in *R. delavayi* varied above-ground tissues under drought stress conditions. From our results, *GAPDH* (*DUH011791.1*) could be recommended as a reference gene for drought stress experiments using developed leaves as the experimental tissue. Furthermore, under control conditions, the topmost stable candidate reference genes at the young stage of *R. delavayi* with developed leaves and stem tips were anticipated to be *UEC1*, *UEC2*, *Actin*, and *GAPDH*, with *UEC1* expected to be the most stable candidate reference gene. qRT-PCR experiments in *R. delavayi* could be improved by combining young and adult organs. TIP41 or GAPDH/their combination could be further validated and used for qRT-PCR experiments in *R. delavayi*. The findings presented here not only identify the most appropriate reference genes for qRT-PCR analysis in *R. delavayi*, but they also provide referable guidelines for other plant species that do not have documented or validated reference genes for qRT-PCR analysis.

## Data Availability

The datasets presented in this study can be found in online repositories. The names of the repository/repositories and accession number(s) can be found in the article/Supplementary Material.
