# Supplementary material for: Selection and Evaluation of Candidate Reference Genes for Quantitative Real-Time PCR in Aboveground Tissues and Drought Conditions in Rhododendron Delavayi
Source: Front Genet. 2022 Apr 14;13:876482. doi: 10.3389/fgene.2022.876482 (PMC9046656; doi:10.3389/fgene.2022.876482)
Supplement: Supplementary file 1 [file Table1.DOCX]

**
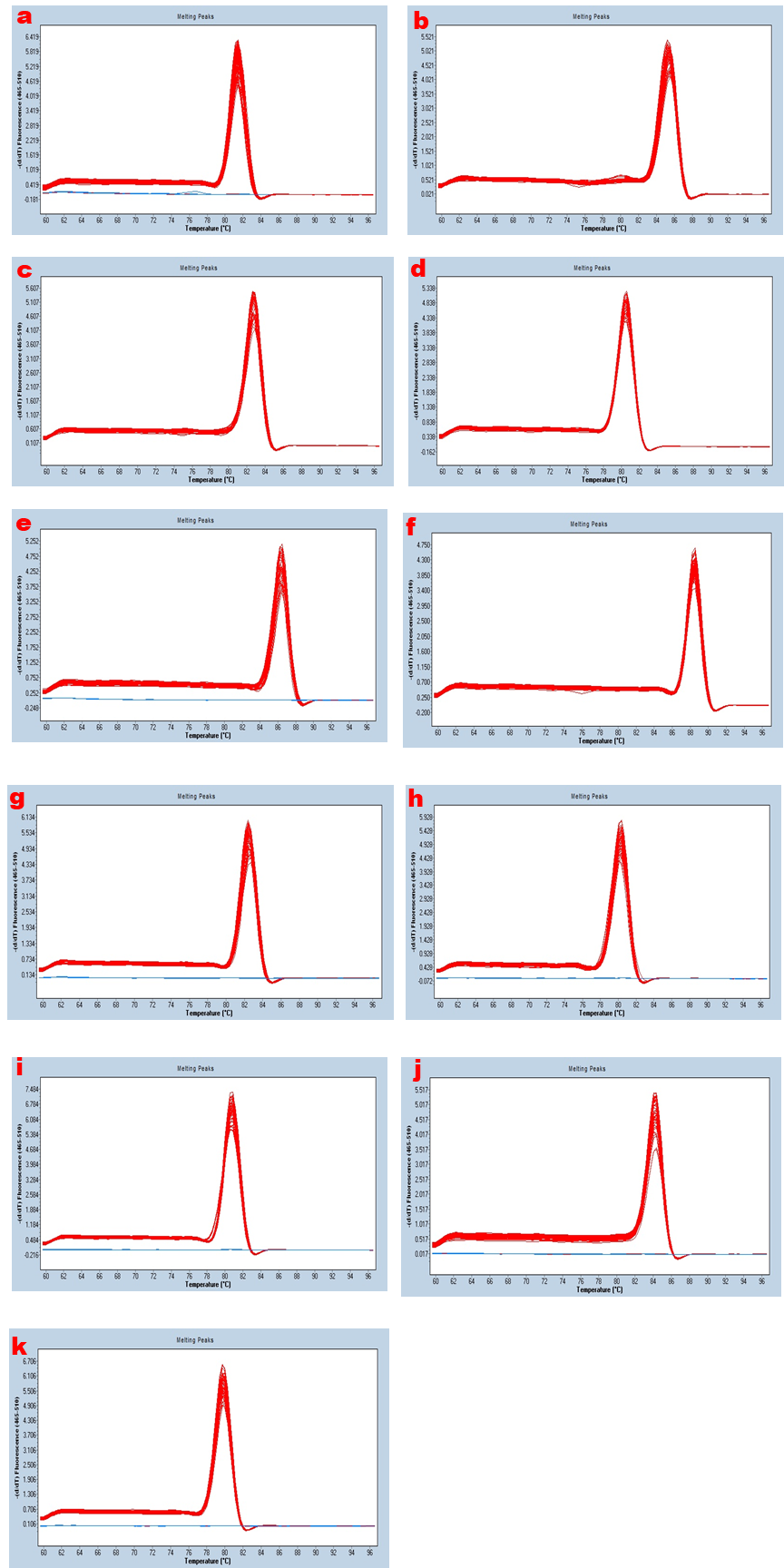
**

**Figure S1.** Melting curves of the eleven candidate genes. a. *GAPDH.* b*. Actin.* c. *EF1α.* d. *Tubulin-β-5.* e. *Ubiqutin.* f. *UEP.* g. *UEC1.* h. *UEC2.* i. *TIP41.* j. *Tubulin-β.* k. *TATA.*
