# Supplementary material for: Selection and Evaluation of Candidate Reference Genes for Quantitative Real-Time PCR in Aboveground Tissues and Drought Conditions in Rhododendron Delavayi
Source: Front Genet. 2022 Apr 14;13:876482. doi: 10.3389/fgene.2022.876482 (PMC9046656; doi:10.3389/fgene.2022.876482)
Supplement: Supplementary file 2 [file Table2.DOCX]

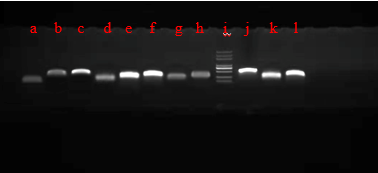


**Figure S2.** Sizes of the eleven candidate reference genes by Agarose gel with 5,000 bp molecular ladder. a. *GAPDH.* b*. Actin.* c. *EF1α.* d. *Tubulin-β-5.* e. *Ubiqutin.* f. *UEP.* g. *UEC1.* h. *UEC2.* i. Molecular ladder (5,000 bp). j. *TIP41.* k. *Tubulin-β.* l. *TATA.*
